# Creating a Conversation: Insights from the Development of a Decision Aid

**DOI:** 10.1371/journal.pmed.0040233

**Published:** 2007-08-07

**Authors:** Victor M Montori, Maggie Breslin, Matthew Maleska, Audrey J Weymiller

## Abstract

The authors share lessons learned from their development of*Statin Choice,* a decision aid for patients with diabetes who are considering using statins to reduce their cardiovascular risk.

Evidence-based medicine requires that clinical decisions be consistent not only with the best available research evidence, but with the values and preferences of the informed patient [1]. To achieve this goal, clinicians and patients can use tools, known as decision aids, that prepare patients for decision making [2] or help clinicians assist patients in participating in making decisions [3].

Our research group has recently completed the development and testing of such a decision aid, dubbed *Statin Choice*, for patients with diabetes who were considering using statins (medications that lower cholesterol) to reduce their cardiovascular risk. *Statin Choice* sought both to help clinicians share the evidence about potential benefits and downsides of statins and to create a two-way conversation that would enable patients to participate in making decisions to the extent they preferred. Patients' participation could make the resulting decisions more likely to be consistent with their values and preferences. In addition, patient participation in decision making (i.e., cognitive investment in the decision and thus “ownership” of that decision) could enhance adherence to therapeutic interventions, among other potential benefits.

Here, we present the insights that resulted from the process we followed to develop this decision aid. In sharing these insights, our intent is not prescriptive (i.e., “this is how you should develop decision aids”), but rather to inspire others to seek innovative, yet goal-directed approaches to the development of decision aids that are not only evidence-based in content but also user-centered in their design and use.

## The Problem

Type 2 diabetes mellitus is a common chronic condition associated with very high costs, both in resources and in human suffering and lives [4]. Cardiovascular events cause most of the diabetes-related deaths and disability, making the reduction of cardiovascular risk a focus of diabetes care [5]. Clinical trials have established that lowering cholesterol with statins can reduce the risk of cardiovascular events in patients with diabetes [6,7]. Current guidelines recommend the use of statins for most patients with type 2 diabetes [8]. Despite the otherwise solid evidence base and the guideline recommendations, few patients take up statins and adhere to these medications over time [9,10].

A model for nonadherence to medications offers some explanations for this phenomenon [11,12], including use of multiple medications, discontinuity of care, and costs of medications. Limited patient knowledge [13] and participation in treatment decision making may also contribute to nonadherence. In particular, clinicians focused on “treating numbers” (e.g., lowering cholesterol to a guideline-directed low-density lipoprotein [LDL] cholesterol goal) may limit the participation of patients who feel unable to make expert judgments about LDL-cholesterol goals and statin dosage [14]. Thus, decision aids that express benefits and downsides in ways patients can understand and value, and the resulting enhanced patient participation in decision making, may improve adherence.

## Some Key Observations

**Figure 1:**
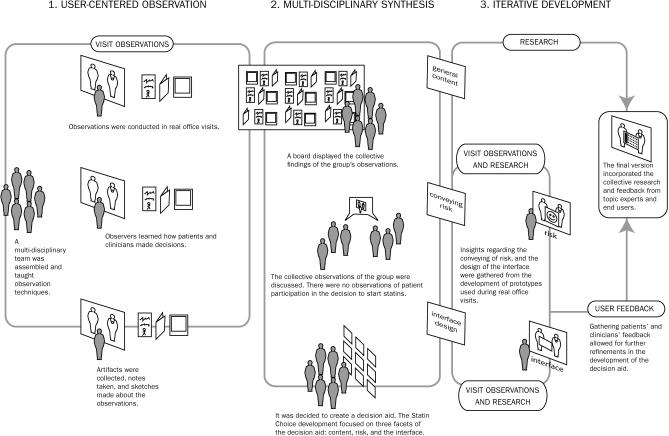
Developing the Statin Choice Decision Aid The design process used to develop our decision aid involved user-centered observation and synthesis and an iterative development process. This process tested versions of the decision aid that incorporated insights from the research evidence, our observations, and user feedback [26].

Figure 1 outlines the design process we used to develop the decision aid. To begin the project, we formed a multidisciplinary team of observers, including clinicians (nurses and doctors) that care for patients with diabetes, patient educators, and administrative personnel. We intentionally invited nonclinicians with little or no knowledge or expectation as to what “normally” happens during diabetes visits with a specialist. After a 30-minute training session about deliberate observation, note taking, illustration (sketching, photography of materials), and “artifact” collection, among other tasks, we asked them to make observations about how patients and clinicians made decisions in a subspecialty setting at a large referral academic group practice (i.e., being a “fly on the wall” at real office visits). The observed parties gave verbal consent; no video or audio recording took place.

As the observers began to populate a board we had assembled for the purpose of collecting artifacts, notes, and photos, and shared stories about their observations with the entire group, it became obvious that there were no stories of patient participation in decision making or descriptions of visits in which clinicians presented the decision to start statins as a choice. Rather, it was apparent that clinicians informed patients about their cholesterol levels and the need to achieve LDL-cholesterol goals (with no mention of a goal to reduce cardiovascular risk), and gave patients a statin prescription. While clinicians offered some instructions as to how to use the medication (e.g., “you should take it at night”), rarely was there a discussion about potential side effects, burden of treatment, cost, or consistency of this tactic with patient goals for life and health, unless patients specifically asked. Taking into account these observations and the theoretical models about nonadherence, we set out to design a decision aid.

## A Proposed Solution—Creating the Conversation

We sought to develop a decision aid that could improve on the one-sided technical decision (which assumes that there is no alternative choice, that the aim is to lower cholesterol levels, that the physician will monitor liver tests and cholesterol levels and will adjust the dose as needed, and that the patient will comply) and that would better acknowledge that patients will make the ultimate decision on adherence, if not explicitly then through their actions (such as never filling the prescription or quitting taking the medication). To develop *Statin Choice*, we focused on three facets of the decision aid: its general content, how it conveys risk, and the design of its interface.

## Content

We first considered the “content” of the decision. This involved determining if the decision to use statins was indeed purely technical. In our view, technical decisions are choices that patients would have limited interest in making because the available options have features and potential outcomes that make little discernable difference, and because if patients were to participate their input would have a limited chance to make an important difference (e.g., the choice of suture material to close the bowel stump after appendectomy). When the decision to use statins is framed as a choice of tactics to lower LDL-cholesterol to a particular goal level, selecting a drug (e.g., statins) and a particular dose appear as purely technical decisions. This is, apparently, the view taken by the guideline developers, the clinicians, the patients, and our observers. As the phrase that one of our observers used suggests (“they had to be on statins”), the decision to take statins is not only perceived as a technical decision, but it is not perceived as a choice. Arguably, evidence of patient nonadherence to statins suggests that patients are making a decision about using statins, but they are not making this choice with their clinician in the office.

To create the conversation, we had to reconsider whether statin use in diabetes is really a technical decision in which patients have no chance to contribute. A recent review found that the research supporting use of statins to lower LDL-cholesterol to specific goals is of very low quality [15]. We thought such low-quality evidence did not support the decisions of guideline developers to make strong recommendations for lowering LDL-cholesterol levels and of quality panels to make the LDL-cholesterol targets of these recommendations quality goals. However, we did find high-quality evidence—from consistent and precise randomized trials [7,16] and from large postmarketing observational studies informing the potential benefits and downsides of statin use [17,18]—supporting the use of statins to lower cardiovascular risk (at a fixed “clinical trial” dose rather than dosing to seek a particular LDL-cholesterol goal). Because patients could meaningfully participate in a discussion about using statins to reduce their cardiovascular risk, we decided that the high-quality evidence supporting such use would inform the content of our decision aid.

## Conveying Risk

To create a useful two-way conversation between patients and clinicians about the decision to use statins, we had to succeed at conveying risk. We had to decide how to represent cardiovascular risk both off and on statins. Focused on the need to offer patients the best chance to participate in the conversation, we decided against presentation formats that would potentially mislead patients in favor of statin use (i.e., presenting relative risk reductions) [19,20]. Several systematic reviews focused on effective risk communication have found that graphs reflecting natural frequencies with a common denominator can convey risk accurately and in a way that patients prefer and understand [21–23]. We had shown that patients with diabetes, even those with low literacy skills, could understand risk presentations that used both words and graphics (e.g., green and happy and red and unhappy ordered faces) [24]. The ordered faces display has the additional advantage of enabling the presentation of information with both positive and negative framing (patients who experienced and patients who did not experience heart attacks).

Our choice of terminology represented a key decision. We avoided phrases such as “your risk of heart attacks in the next ten years…” since it is not possible to confidently estimate a given individual's risk from group data, and because that individual either will or will not have the heart attack in the next ten years (risk of 0% or 100%). We needed an accurate yet simple story to tell patients about risk. After several iterations, we came to:

*“Imagine you are in a room with 100 patients like you for the next 10 years. At the end of that period you look back and you find X people who have had a heart attack and 100 - X that have not. Notice that those who had the heart attack did so at some point during the 10 years, and that we cannot tell when someone will have a heart attack. Also note that we cannot tell whether you are one of those destined to have a heart attack (displayed using red and unhappy faces) or not (displayed using green and happy faces) in the next 10 years...”*

To represent the absolute risk reduction with statins, we created a parallel presentation for a group of “100 patients like you” who took statins for ten years. In this version of the story, it was important to note that only a small proportion of patients destined to have heart attacks could avoid this outcome by taking medication and that statins cannot change the prognosis of the majority of patients. To clarify this key point, we added:

*“Notice that we cannot tell whether you will be one of the green ones (patients who were not destined to have heart attacks but took medication anyway), yellow ones (patients destined to have a heart attack but who avoided one by using statins) or red ones (patients who suffered a heart attack despite taking statins).”*

We created similar content to convey the risk of potential side effects of statins (see decision aid at http://mayoresearch.mayo.edu/mayo/research/ker_unit/decision-aids.cfm), with attention to the use of plain language [25].

## The Interface

At least as important as the decisions we made about the content of the decision aid (enabling choice, based on high-quality evidence, quantitative, and both positive and negative framing) were decisions about the interface between the users and the decision aid.

Early in our development process [26], we invited key informants (patient education specialists, clinicians, diabetes educators, and patients) to brainstorm about the ideal interface for our decision aid. It became clear that there was a strong preference for interacting with the decision aid through an electronic interface (e.g., personal, portable, or desktop computers accessing interactive DVDs or the Internet). Some reasons for this included the ability to tailor the information according to the user, the possibility of updating the information easily, and the portability of the material, particularly if the material was placed and accessed on the Internet.

When asked to review a draft version of the decision aid we posted online, clinicians thought that this electronic version would allow the patient to use the decision aid without supervision, perhaps at home, in preparation for the visit. Patients, on the other hand, found themselves printing out the material to share its content with significant others. An online decision aid seemed then likely not to have a meaningful impact on the conversation; furthermore, the format was not sufficiently portable for patients who wanted to share it with other important parties to the decision. Also, this approach would place the onus of creating a conversation almost entirely on patients. Confronted with the expertise and authority of the clinicians, most patients may not feel comfortable enough to bring up concerns or questions that the decision aid may have provoked. From this insight, we decided that the decision aid needed to be present in the exam room during the consultation in order to have a chance at creating a conversation.

With the ideas of portability and presence in the exam room, we looked to booklets. Booklets could capture the information we wanted to share and have been used extensively to educate patients at Mayo Clinic. Interestingly, both patients and clinicians did not think the booklet would be efficient enough to use during the visit, and clinicians thought patients would have to review the material ahead of time. At this point we decided to try a one-page version. Several such versions were tried until, through a process of elimination and simplification of the information, we arrived at a version that clinicians were willing to use during the visit with patients and that patients felt was a helpful snapshot of the issues of importance for the decision (see final decision aid at http://mayoresearch.mayo.edu/mayo/research/ker_unit/decision-aids.cfm).

Our confidence in this version was reinforced by the fact that a conversation quite different from those observed earlier was started when clinicians and patients tried using the one-page tool. If for no other reason, conversations were started to complete the information presented in the necessarily abbreviated and incomplete one-page version. Furthermore, use of a one-page tool required minimal clinician training in using the tool, offered extreme portability, and was not expensive to produce. Another key point is that the “bare bones” nature of the decision aid and its nondirective tone allowed clinicians the flexibility to incorporate the decision aid within the workflow of their visits, to use it within their own communication and decisional styles, and to express their own preferences.

A paper-based tool did not offer many opportunities for real-time tailoring. Thus, we created versions for patients at three different levels of risk. Each decision aid had the patient's name, level of risk (estimated using specific patient characteristics [27]), list of interventions already in place that could reduce the risk of cardiovascular disease, the risk of cardiovascular events off and on statins, a quantitative description of potential harms and inconveniences, and a question seeking to determine whether the patient was ready to participate in a decision now or would prefer to delay the decision. The final version also had graphical links with a more complete booklet; these links enabled the patient to complete the information in the one-page aid with the detailed descriptions in the booklet outside of the consultation.

The one-page tool does not include a graphical representation of the risk for downsides of therapy (the graphs are included in the booklet). A common temptation is to show the risk for downsides in the efficacy graphs, rather than presenting graphs for each downside. As an unintended consequence, this presentation conveys the message that side effects and heart attacks (those experienced and those avoided by therapy) are mutually exclusive outcomes. That is, patients might assume that if the medication gave them elevated liver enzymes or muscle aches then they would neither get nor avoid heart attacks. The design of the one-page decision aid trusted that the resulting conversation would help patients contextualize the benefits in terms of the potential downsides.

## The Result

We conducted a 98-patient clustered randomized trial of *Statin Choice* versus a standard educational pamphlet in patients with diabetes attending a subspecialty clinic [28]. A full description of the study and its results is available at http://kerunit.e-bm.org. We found that patients expressed a strong preference for the decision aid and a majority recommended it for other patients considering the same choice. The majority also requested a similar tool for future important decisions. The decision aid also proved effective in communicating information about pros and cons and in communicating risk and absolute risk reduction. This, in turn, reduced patient uncertainty about the best course of action and increased the proportion of patients who adhered to statins at three months [28].

Our analyses of the videotaped encounters in this trial substantiate the observation that, in most cases, the decision aid helped create a conversation about statins. Using the OPTION scale, a validated scale that quantifies the extent to which clinicians involve patients in decision making [29], on the videotapes, we found substantial increase in the degree of patient involvement in decision making whenever the decision aid was used during the visit.

The resulting decision aid satisfied international consensus-based standards for decision aids [30]. However, as with most complex interventions, it is difficult to measure the relative contributions of each of the design features and their interaction. Large samples and design permutations in content, interface, and implementation would be necessary. Our approach has been pragmatic: let the evidence accumulated thus far about effective risk communication and early and frequent user input guide the design process. The results up to this point support this approach.

Furthermore, it is striking that our results are consistent with the experience of other developers who have learned, through different empirical and experiential approaches, the value of preserving flexibility, optimizing the information content and interface, determining the appropriate technology for the purpose, and ensuring patient and clinician input throughout the process to enhance its acceptability [31,32].

In summary, we designed the decision aid *Statin Choice* to help clinicians create a conversation about medications with patients with diabetes. This new conversation is more patient-centered and evidence-based, and therefore more likely to achieve the goal we pursued: that the decisions made during the visit be more consistent with the evidence and the values and preferences of the informed patient.

## Abbreviations

LDL: low-density lipoprotein
